# Cyclic fatigue resistance of a blue heat-treated engine-driven file with different angles of canal access using different kinematics; an *in vitro* study

**DOI:** 10.3389/fdmed.2025.1636746

**Published:** 2025-08-26

**Authors:** Bassem Eid, Mohammed Kataia, Tarek Elsewify

**Affiliations:** ^1^ Restorative Dental Science Department, College of Dentistry, Gulf Medical University, Ajman, United Arab Emirates; ^2^ College of Dentistry and Health Sciences, Fujairah University, Fujairah, United Arab Emirates; ^3^ Restorative Dental Science Department, College of Dentistry, Gulf Medical University, Ajman, United Arab Emirates; ^4^ Endodontic Department, Faculty of Dentistry, Ain Shams University, Cairo, Egypt

**Keywords:** angle of access, dynamic cyclic fatigue, E3 azure, rotation, reciprocation, time to fracture

## Abstract

**Objectives:**

To evaluate the effect of angle of access and kinematics on the dynamic cyclic fatigue resistance of E3 Azure rotary NiTi files at body temperature.

**Methods:**

Eighty E3 Azure files, 25/06, were randomly divided into two equal groups according to the kinematics used, rotation, and reciprocation. Each group was further divided into two equal subgroups (*n* = 20) according to the angle of file access, zero degrees and 30 degrees. The files were placed in custom-made stainless-steel canals and operated using the manufacturer's recommendations for speed, torque, and angle of reciprocation settings till fracture. The time to fracture and the fracture length were measured. Statistical analysis was performed at a significance of 0.05.

**Results:**

Samples instrumented using reciprocation motion had significantly higher time to fracture than those with continuous rotation (*p* < 0.001). Samples with zero-degree access angle had significantly higher time to fracture than those with 30° (*p* < 0.001).

**Conclusion:**

The motion and the angle of file access significantly influence the cyclic fatigue resistance of E3 Azure files. Reciprocation motion and a smaller angle of access improve the fatigue resistance of E3 Azure files.

**Clinical relevance:**

Reciprocation motion and establishment of straight-line access enhance the safety and efficiency of E3 Azure files.

## Introduction

The modified conservative access cavities have been the scope of interest for all bio-minimalistic researchers since 2018 (1). Minimally invasive endodontic treatment aims to preserve as much of the natural tooth structure as possible while ensuring effective debridement and disinfection of the root canal system. The contracted access cavity is a key component of minimally invasive endodontics, characterized by a conservative access design that minimizes removal of pericervical dentin and maintains the structural integrity of the tooth. The problem with contracted cavities is that they place the file under high stress due to the absence of a straight path for the enlarging tool into the canal (2).

Recent heat-treated files having higher cyclic fatigue resistance have been developed (3). Among these instruments, the E3 Azure files (Endostar, Zielonka, Poland), characterized by their unique design and material properties, have garnered attention for their performance and durability (4). The Endostar E3 Azure is an advanced endodontic system designed using Azure HT Technology, a specialized heat-treatment manufacturing process. This innovative technology enhances the flexibility and fracture resistance of the files, making them highly reliable even in complex clinical cases. The Azure HT Technology process alters the crystal structure of nickel-titanium files, enabling the transformation from martensite to austenite to occur near body temperature. The E3 Azure features a modified S-shaped cross-section, which reduces the file's core size to enhance debris removal and improve flexibility. It also incorporates a variable pitch, neutral rake angle, and a safe cutting tip for optimal performance (5).

In addition, recent kinematics were proposed as an alternative for continuous rotation and allowed increased time to failure for the instrument (6, 7). The reciprocating motion refers to the use of alternating clockwise and counterclockwise movements during root canal instrumentation. This motion reduces torsional stress on the file and the risk of instrument separation, especially in curved or narrow canals. The reciprocation motion has been tested through a lot of different factors and compared to rotational cutting motions in multiple aspects (6, 8–10).

The manufacturer's recommendation for the E3 Azure file is the use in a full rotation motion, a reciprocation motion, or a complex motion, which is comparable to the adaptive motion proposed earlier by Kerr for the TF adaptive file system. The file design, specifically, the neutral rake angle, allows for the use of this file in variable motions (6, 8–10).

The angle of file access into the root canal plays a crucial role in determining the cyclic fatigue resistance of endodontic instruments (11). When a file enters the canal at a sharp angle, it is subjected to increased flexural stress, particularly at the point of maximum curvature. This repeated stress accumulation accelerates crack initiation and propagation within the metal structure, ultimately reducing the file's lifespan and increasing the risk of separation. Therefore, maintaining an optimal file access angle through proper access cavity design and glide path preparation is essential in prolonging instrument longevity and ensuring efficient root canal shaping (5, 6, 12).

The 0° and 30° angles of file access are representative of conventional and contracted endodontic access cavities, respectively, because they simulate the straight-line path of an instrument entering the canal through differently designed access outlines. A 0° angle reflects the file being inserted along the long axis of the tooth, which is typically feasible in conventional access cavities due to total removal of the roof of the pulp chamber and a straight-line access to the apical foramen or the initial canal curvature. In contrast, a 30° angle mimics the angulated approach required in contracted and conservative access cavities, which necessitates an indirect or more oblique entry point due to preservation of the coronal dentine and lack of straight-line access to the canal (5, 12).

However, the combined effect of access cavity angulation and kinematics on the dynamic cyclic fatigue resistance of recently introduced heat-treated NiTi files, such as E3 Azure, has not yet been investigated. Furthermore, most available studies do not simulate clinically relevant conditions, such as dynamic movement (9) and body temperature (9, 12), limiting their applicability and clinical relevance.

Therefore, this study aims to assess the influence of angled canal access on file separation, along with the impact of different cutting motions. The null hypothesis states that there is no significant difference in the time to file separation between full rotation and reciprocation motions when the access angle is either 0° or 30°.

## Materials and methods

### Ethical approval

The research proposal was revised and approved by the institutional review board, Gulf Medical University, Ajman, UAE, IRB-COD-FAC-49-Oct-2024.

### Sample size calculation

A power analysis was performed based on the results obtained by La Rosa et al. (13) who evaluated the effect of access angle and temperature on the cyclic fatigue resistance using the G-power program 3.1.9.4 by adopting an alpha error of 0.05, power of 0.8, and an effect size of 1.02. The predicted sample size was found to be 80 (*n* = 20).

### Sample grouping

The samples (*n* = 80) were divided into two main groups (each *n* = 40),

Group I: instruments were used in continuous rotation motion.

Group II: instruments were used in reciprocation motion.

**Figure 1:**
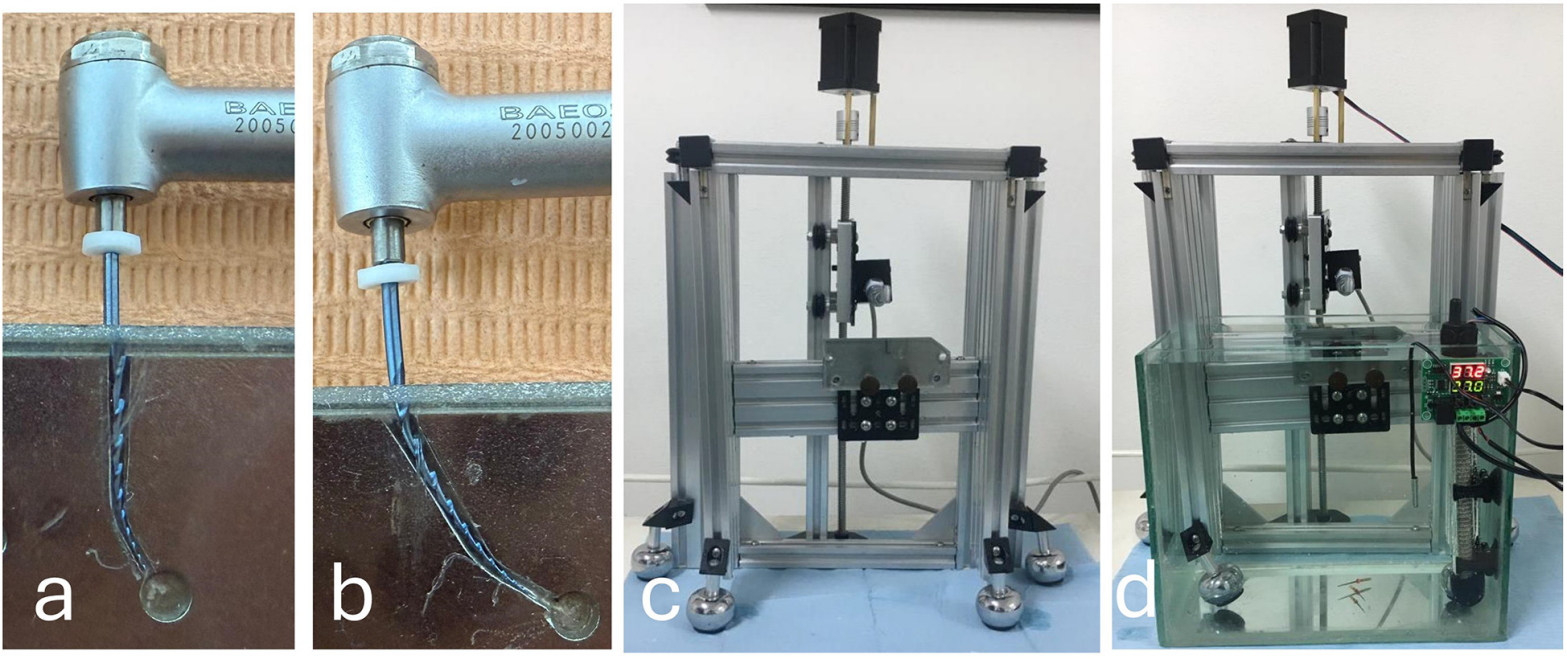
Dynamic cyclic fatigue testing for E3 azure file **(a)** access angle of 0°; **(b)** access angle of 30°; **(c)** frontal view of the testing device; **(d)** cyclic fatigue testing device in water bath at body temperature.

Each group was further subdivided into 2 subgroups:

Subgroup I A: where the instrument operated with an access angle of 0^o^ (Figure 1a).

Subgroup I B: where the instrument operated with an access angle of 30^o^ (Figure 1b).

### Dynamic cyclic fatigue testing

A total of 80 E3 Azure heat-treated NiTi files, 25/06, were randomly divided into 4 equal groups (*n* = 20) using a random sequence generated by Microsoft Excel. All the files were inspected using the dental operating microscope (Global Surgical Corporation, Saint Louis, MO, USA) after unboxing to confirm the absence of any defects. Time-to-fracture was tested in a dynamic motion at body temperature using a dynamic cyclic fatigue testing device, which has been used and explained previously (14, 15). The device consisted of a custom-fabricated linear actuator mounted on a specially designed frame that secured both the handpiece and the artificial canal. A custom code regulated the vertical movement 1.5 mm upward over 0.5 s and 1.5 mm downward over another 0.5 s (15).

The same methodology was adopted to maintain the body temperature by performing the testing in a water bath. The dynamic motion was created by a 3 mm vertical motion at a one Hertz frequency till file separation occurred. The time-to-fracture was measured in seconds using a digital stopwatch, and the fractured segment was measured in millimeters using a digital caliper.

Two custom-made artificial stainless-steel canals were designed using AutoCAD software (Autodesk, San Francisco, California, USA), with a tip diameter of 0.25 mm and a taper of 6%, incorporating a 0.1 mm circumferential relief. The canals measured 16 mm in length. Both canals showed the same angle and radius of curvature of 60 degrees and 3 mm; yet they differ only in the angle of access, being zero degrees and 30 degrees, as clearly shown in Figure 1 (5, 14, 15). Stainless steel blocks were milled using a milling machine following the design.

The instruments were used in accordance with the manufacturer's recommendations, using E-Connect S endodontic motor (Eighteeth, Jiangsu Province, China). In the rotational mode group, the instruments were operated at a speed of 300 rpm with a torque of 3 N/cm, while the reciprocation group instruments were operated with a clockwise angle of rotation of 120 degrees and 30 degrees counterclockwise rotation.

### Statistical analysis

Numerical data were presented as mean and standard deviation (SD) values. They were tested for normality and variance homogeneity by viewing distribution and using Shapiro–Wilk's and Levene's tests, respectively. The data were normally distributed with homogenous variances across different variables. They were analyzed using a two-way ANOVA test. The comparisons of simple effects were made using the error term of the two-way model. *P*-values were adjusted for multiple comparisons using the False Discovery Rate (FDR) method. The significance level was set at *p* < 0.05 within all tests. Statistical analysis was performed with R statistical analysis software version 4.4.1 for Windows (R Development Core Team, Auckland, New Zealand).

## Results

The two-way ANOVA results presented in Table 1 showed that there was a significant interaction between both tested variables (*p* < 0.001). The simple effects comparisons presented in Table 2 showed that regardless of access angle, samples instrumented using reciprocation motion had significantly higher time to fracture than those with continuous rotation (*p* < 0.001). Additionally, regardless of rotation motion, samples with zero-degree access angle had significantly higher time to fracture than those with 30° (*p* < 0.001). Mean and standard deviation values for the fracture-segment length were statistically not significant, as presented in Table 3.

**Table 1:** Two-way ANOVA results for multiple comparisons.

Source	Sum of squares (III)	df	Mean square	f-value	*p*-value
Rotation	289,595.31	1	289,595.31	7,007.22	<0.001*
Angle of entry	1,136,869.81	1	1,136,869.81	27,508.38	<0.001*
Rotation* angle	6,310.13	1	6,310.13	152.68	<0.001*

df degree of freedom.

* Significant (*p* < 0.05).

**Table 2:** Simple effects comparisons of the kinematic and angle of file access.

Angle of entry	Time to fracture (seconds) (Mean ± SD)		f-value	*p*-value
	Continuous rotation	Reciprocation		
0°	548.98 ± 6.19	719.15 ± 5.80	7,007.22	<0.001*
30°	211.80 ± 5.72	346.45 ± 7.78	4,386.99	<0.001*
f-value	27,508.38	33,610.35		
*p*-value	<0.001*	<0.001*		

* Significant (*p* < 0.05).

**Table 3:** Simple effects comparisons of the fracture-segment length.

Angle of entry	Fracture-segment length (mm) (Mean ± SD)		*p*-value
	Continuous rotation	Reciprocation	
0°	3.312 ± 0.04142	3.3075 ± 0.04856	*p* = 0.98745
30°	3.3045 ± 0.04641	3.315 ± 0.030248	*p* = 0.86748
*p*-value	*p* = 0.94610	*p* = 0.94610	

## Discussion

Several factors contribute to file fracture; however, cyclic fatigue has been identified as a major cause, particularly when instruments are used in curved root canals (16, 17). Dynamic cyclic fatigue testing, despite its limitations in replicating the full complexity of clinical conditions, remains one of the most reliable and standardized methods for evaluating the fatigue resistance of endodontic files (6, 18, 19). Unlike static models, dynamic testing incorporates axial movements that closely mimic the clinical pecking motion, resulting in more realistic stress distribution along the instrument (18). This approach allows for controlled and reproducible comparisons between instruments, motions, and access designs, while isolating variables such as file design, kinematics, and access angle. In the present study, a custom-made stainless steel artificial canal with a 60° curvature and 3 mm radius was used to simulate clinical conditions (5, 11). The dynamic model better replicates intracanal stresses by distributing them along the file shaft, rather than concentrating stress at a single point, as observed in static models (20).

The temperature changes significantly influence the cyclic fatigue resistance of heat-treated endodontic NiTi files. An increase in temperature promotes phase transformation to a more austenitic form, which reduces cyclic fatigue resistance and accelerates microcrack propagation (21, 22). The test was conducted at body temperature to most accurately replicate clinical conditions.

Endodontic files entering the canals at steeper angles, the stress concentration at the point of maximum curvature significantly increases, leading to a higher incidence of instrument fracture. For instance, studies utilizing artificial canals and simulated clinical conditions have reported that files subjected to greater access angle experience a marked reduction in the number of cycles to failure compared to those with a more aligned entry path (6).

This study evaluated the effects of kinematics and access angle on the cyclic fatigue resistance of blue heat-treated NiTi files in simulated root canals. The methodology was validated by the nonsignificant difference in fractured segment lengths across all groups (f-ratio = 0.23414), with fractures consistently occurring at the canal curvature, confirming cyclic fatigue as the failure mode. As all files fractured in a predictable and uniform manner, and since only one file system (E3 Azure) was tested without variation in design or metallurgy, scanning electron microscopy was not performed, as it would not have provided additional insights (18, 19).

The results of this study demonstrated that both the angle of access and the type of motion significantly influence the dynamic cyclic fatigue resistance of blue heat-treated NiTi files; therefore, the null hypothesis tested was rejected.

Reciprocating motion demonstrated significantly longer time to fracture than continuous rotation, likely due to its alternating movement, which allows periodic stress release and reduces crack initiation and propagation. The unequal forward and reverse angles in reciprocation help minimize continuous tension and compression cycles, delaying fatigue failure. In contrast, continuous rotation imposes constant stress at the same points, accelerating crack development. This finding supports previous studies reporting enhanced cyclic fatigue resistance with reciprocation due to stress relief from rotational reversal (6, 23). However, some studies found no significant difference, possibly due to variations in file design (9), or modifications in the reciprocation motion (24).

In addition, the results of this study showed that the access angle affected cyclic fatigue resistance, as the files with a zero-access angulation showed significant resistance to cyclic fatigue in comparison with the 30° access angle. This could be attributed to the minimal magnitude of flexural stress imposed on the file. When the file enters straight into the canal without angulation, the distribution of cyclic stress is more uniform along its length, reducing the likelihood of localized stress concentration. In contrast, a 30-degree access angle introduces additional bending forces, increasing strain at specific points along the file, particularly at the curvature, which accelerates crack initiation and propagation. This increased mechanical stress leads to faster fatigue failure, reducing the instrument's lifespan (5, 11).

The findings of this study align closely with those of Assaf et al. (5), who also studied E3 Azure files in comparison to OneCurve files in full rotational motion. Their study demonstrated that a smaller curvature angle and a more coronal curvature position enhanced the fatigue resistance of the tested nickel-titanium rotary files.

Pedullà et al. (12) results align with our results evaluating the cyclic fatigue resistance of Reciproc and Reciproc Blue files at various angles of file access in a reciprocation motion. The smaller the angle of access, the higher the cyclic fatigue resistance of both tested files.

Despite their value, *in vitro* studies using simulated canals at body temperature have limitations. Artificial models cannot fully replicate the complexity of natural teeth, including anatomical variations, dentin properties, and clinical factors like debris, lubrication, and operator technique. While body temperature testing is more relevant than room temperature, it still fails to capture dynamic intraoral conditions. Therefore, caution is needed when applying these results clinically, and future research using *in vivo* studies and advanced simulations, such as finite element analysis, is recommended to better reflect real-world performance (18, 19).

There is a growing need for a more standardized and clinically relevant method of measuring the cyclic fatigue resistance of endodontic rotary instruments. Future research should focus on developing advanced testing protocols that incorporate realistic anatomical variations, simulated body temperature, and dynamic loads to better predict clinical performance. Additionally, integrating high-resolution imaging and finite element analysis could provide deeper insights into crack initiation and propagation, ultimately improving instrument design and clinical safety (19).

In conclusion, both the mode of motion and the angle of file access significantly influence the cyclic fatigue resistance of endodontic instruments. Reciprocation motion, which alternates clockwise and counterclockwise movements, reduces stress accumulation and delays crack propagation, thereby enhancing file longevity compared to continuous rotation. Additionally, a smaller access angle minimizes excessive flexion and stress concentration at the point of maximum curvature, further improving fatigue resistance.

These findings emphasize the importance of optimizing both motion kinematics and straight-line access to root canals to enhance the safety and efficiency of endodontic instrumentation.

## Data Availability

The raw data supporting the conclusions of this article will be made available by the authors, without undue reservation.
